# The Effect of Surface Characteristics of Clays on the Properties of Starch Nanocomposites

**DOI:** 10.3390/ma15217627

**Published:** 2022-10-30

**Authors:** Erika Fekete, Lilla Angyal, Emília Csiszár

**Affiliations:** 1 Institute of Materials and Environmental Chemistry, Research Centre for Natural Sciences, Magyar Tudósok Körútja 2, H-1117 Budapest, Hungary; 2 Laboratory of Plastics and Rubber Technology, Department of Physical Chemistry and Materials Science, Faculty of Chemical Technology and Biotechnology, Budapest University of Technology and Economics, Műegyetem rkp. 3, H-1111 Budapest, Hungary

**Keywords:** starch/clay nanocomposite, laponite, montmorillonite, mechanical properties, structure, reinforcing effect

## Abstract

In this research, different clays such as laponite and montmorillonite (NaMMT) are used as fillers in the preparation of thermoplastic starch/clay nanocomposites. Thin films are produced by casting and evaporation in a wide composition range, using glycerol as the plasticizer at two different concentrations. The surface energy of clay fillers is measured by inverse gas chromatography (IGC); X-ray diffraction (XRD) and light transmission measurements (UV-VIS) are carried out to characterize the structure of nanocomposites; and mechanical properties and water vapor permeability are also studied. While all the starch/montmorillonite nanocomposites possess intercalated structures, significant exfoliation can be noted in the starch/laponite nanocomposites, mainly at low clay contents. Due to the larger surface energy of montmorillonite, stronger polymer/clay interactions and better mechanical properties can be assumed in starch/NaMMT composites. The smaller surface energy of laponite, however, can facilitate the delamination of laponite layers. Thus, the specific surface area of laponite can be further increased by exfoliation. Based on the results, the better exfoliation and the much larger specific surface area of laponite lead to higher reinforcement in starch/laponite nanocomposites.

## 1. Introduction

Polysaccharides are promising biopolymers that are widely used in the packaging, agricultural, healthcare, and medical areas as an alternative to petroleum-based polymers. Films, nanoparticles, gels, and scaffolds are usually developed from these biopolymers [1,2,3,4,5,6]. Starch is one of the best-known and most commonly used polysaccharides because it is cheap and available in large quantities. In the preparation of plastic products from starch, granular starch is processed in the presence of plasticizers. Glycerol and water are usually used for this purpose, and the final thermoplastic starch (TPS) can be prepared by thermomechanical methods (extrusion, injection molding) or from solution by film casting. The main disadvantages of TPS are its hydrophilicity and inadequate mechanical properties, which can be improved by blending TPS with other polymers or fillers [7,8,9,10,11,12,13,14,15].

Polymer/clay nanocomposites are assumed to exhibit improved barrier, thermal, and mechanical properties compared with traditional composites. Recently, researchers have reported in the literature several attempts at preparing TPS nanocomposites. In most cases, TPS/montmorillonite (NaMMT) nanocomposite films were prepared by melt blending or by film casting. The results demonstrated that the incorporation of NaMMT results in an intercalated/exfoliated structure of TPS nanocomposites [16,17,18,19,20,21]. In addition to the type of the components, the ratio of intercalated and exfoliated clay particles is significantly influenced by the amounts of plasticizer and NaMMT due to the competitive interactions among the polymer, plasticizer, and clay [16,17,18,22,23,24,25,26]. Our results [20], in a similar way to others published in the literature [18,22,23,24,25,26,27], proved that although the exfoliation is never complete, the TPS/NaMMT nanocomposites have improved properties compared to TPS. The nanocomposites’ properties strongly depend on the type of starch and montmorillonite used, as well as the amounts of NaMMT and glycerol. Papers published so far indicate that a more significant extent of exfoliation results in better properties [18,22,23,24,26,27]. In addition to their barrier properties, packaging materials should also possess good mechanical characteristics. Although several papers discuss the stiffness, strength, and deformability of TPS nanocomposite films [19,20,22,23,24,26,27,28], only a limited number of papers report systematic experiments carried out as a function of filler content in a wide composition range [20,24,28].

Since NaMMT is a natural mineral produced by mining, it usually contains contaminants and irregularities in its crystalline structure, making it necessary to explore and find other silicate fillers that do not have these drawbacks. A synthetic layered silicate, laponite, is an obvious alternative because its chemical and crystalline structures are well known. Laponite is a synthetic silicate (Laponite XLG composition: Na+0.7[(Si8Mg5.5 Li0.3)O20(OH)4]-0.7) with the same structure as the clay mineral hectorite, another smectite similar to NaMMT. Compared to natural NaMMT, synthetic laponite presents more well-defined compositions and is free of impurities and accessory minerals. The individual laponite particles have a more regular disk shape, and their lamellae are smaller than natural NaMMT lamellae, with an average diameter of 25 nm and thickness of 1 nm [29]. Our former study [30] proved that the specific surface area and surface energy of NaMMT and laponite differ significantly.

Since the properties of polymer composites strongly depend on the specific surface area of the filler and the polymer/filler interfacial interaction [9,11,15,20], it would be interesting to know how these surface properties of NaMMT and laponite influence the structure and properties of the TPS nanocomposites of these clays.

Although laponite is also a popular clay nowadays, there are few publications on TPS/laponite nanocomposites [29,30,31,32,33,34,35]. In these publications, the authors focus primarily on the microstructure of TPS/laponite nanocomposites. Although it is established that TPS/laponite nanocomposites show a similar exfoliated/intercalated structure to that of TPS/NaMMT composites, and laponite also improves the mechanical, thermal, and barrier properties of TPS, the reported results are not convincing in all cases. Moreover, the authors did not investigate the role of interfacial interactions on the composite properties.

Unfortunately, there is also no publication in the scientific literature that compares the effects of NaMMT and laponite on TPS nanocomposite properties, though the effects of the two fillers have been compared for several other polymers [36,37,38,39,40]. It is not easy to draw any conclusions from the published results, since the effect of laponite and montmorillonite in different polymers depends not only on the polymer type but also on the preparation methods used. Therefore, the goal of the present work was to study the effect of the specific surface area and surface energy of laponite and montmorillonite on the structure and properties of TPS nanocomposites.

## 2. Materials and Methods

### 2.1. Materials

High-quality corn starch produced in Hungary (Hungrana Ltd., Szabadegyháza, Hungary) was used in the experiments. Its water content was 12%. Glycerol with a 0.5 wt% water content was obtained from Sigma-Aldrich Kft. Hungary and was used without further purification or drying.

Two kinds of layered silicate were studied during the experiments: sodium montmorillonite (Nanofill 116) supplied by Southern Clay Products (Rockwood Additives Ltd., Gonzales, LA, USA) and Laponite XLG obtained from Byk Additives and Instruments (Wesel, Germany). The most important characteristics of the silicates are summarized in Table 1.

### 2.2. Preparation of TPS Nanocomposite Films

TPS nanocomposite films were prepared by the solution casting method [17,20]. Solution mixing was carried out using the following method. Native starch was dispersed in the excess amount of distilled water. The suspension was continuously stirred at 80 °C for 20 min to gelatinize the corn starch granules. The starch concentration of the solution was 4.5 wt %. Sodium montmorillonite (NaMMT) and Laponite XLG were dispersed in distilled water at a concentration of 0.8 wt% by sonication for 20 min at room temperature. The clay dispersion was added to the aqueous gelatinized starch, and the mixture was stirred for another 20 min at 80 °C. Finally, 30% or 40% glycerol (relative to the mass of starch) was added to the starch/clay suspension, and it was stirred for another 20 min at 90 °C. The composition and designation of TPS samples are listed in Table 2.

The films were obtained by casting the hot suspension in Petri dishes covered by a Teflon sheet and dried in a climate chamber at 35 °C for 24 h at 50% RH. The clay content changed between 0 and 20 wt % based on the amount of dry starch. The thickness of the films was 0.10 ± 0.02 mm. Before testing, the films were stored at 23 °C and 50% RH (in a climate chamber) until a constant weight was reached.

### 2.3. Characterization

The crystalline structure of the TPS and its composites was studied by X-ray diffraction (XRD) using Philips PW1830/PW1050 equipment with Cu Kα radiation at 40 kV and 35 mA. Samples were scanned in the diffraction angle range of 2–15° in 0.04° steps. Diffractograms were recorded on the powders (starch) or films using a multipurpose sample stage. The basal spacing of the silicate layers was calculated using Bragg’s equation. The light transmission of films at 700 nm was determined using a UV-VIS spectrometer (UnicamW500). Tensile properties (Young’s modulus, tensile strength, and elongation at break) were measured by Instron 5566 apparatus according to ISO 527/4A. The Young’s modulus was determined at 0.5 mm/min while ultimate properties were determined at a cross-head speed of 5 mm/min. All characteristics were derived from five parallel measurements.

The water vapor permeability (WVP) of films prepared by casting was investigated at a 100–50% relative humidity difference. The film was placed between two metal rings on top of a glass cell containing distilled water, and then the glass cell was introduced to a climate-controlled chamber regulated at 50% RH and 23 °C. The test cell was periodically weighted to a constant variation rate of weight. WVP (g·m^−1^·s^−1^·Pa^−1^) was calculated using Equation (1), where m is the weight loss (g) of the test cell, x is the film thickness (m), and A is the exposed area (m^2^) during Δt duration (s) under Δp partial water vapor pressure (Pa).

WVP = Δm x/A Δt Δp(1)

**Figure 1:**
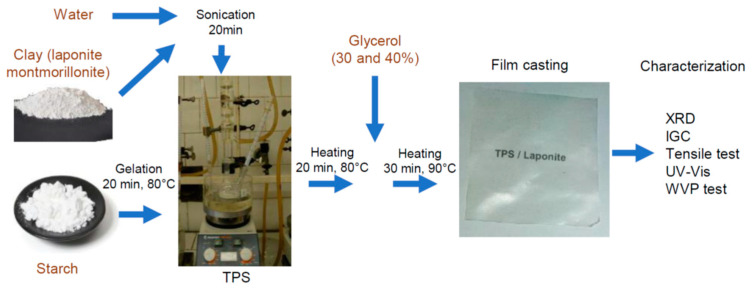
Treatments applied to prepare TPS/nanoclay films from starch powder and montmorillonite/laponite. A schematic representation of the multi-step process.

The dispersion component of the surface tension (γ_s_^d^) of the clay samples was determined by inverse gas chromatography (IGC) at an infinite dilution using n-alkanes with different chain lengths. IGC measurements were carried out using Perkin Elmer Autosystem XL apparatus at 160 °C without conditioning the samples. A new column was prepared for each set of experiments. Vapor samples of 5–20 μL were injected into the column, and an FID detector recorded retention peaks. Each reported value is the result of three parallel measurements. High-purity nitrogen was used as the carrier gas, the flow rate of which changed between 40 and 60 mL/min depending on the types of adsorbent and adsorbate. The γ_s_^d^ values were calculated by the method put forward by Dorris and Gray [42]. Figure 1 shows the preparation steps and methods used in the research.

## 3. Results

### 3.1. Surface Energy of Clays

It is well-known that the properties of polymer composites strongly depend, among other factors, on the component properties and the interfacial interactions, which are functions of the surface energy of the components [43]. Determination by IGC of the surface properties of fillers with extremely high surface energy is difficult because the retention time of the probes applied during measurements can be very long, and they can only be studied at high temperatures. In addition, these fillers always adsorb water on their surface, which modifies their surface energy. That is why conditioning at 100 °C is usually applied before the measurement. In this study, however, the nanocomposites were prepared in water, so interaction was developed between the plasticized starch and the wet fillers. Just the surface energy of the wetted clays was required for the calculation, so no conditioning was applied. Table 3 shows the retention times measured on the clays and the dispersion component of surface tension calculated from them using the Dorris–Gray method.

From Table 3 it is clear that although the surface tension of NaMMT is more significant than that of laponite, the retention times of n-alkanes are much longer on laponite than on NaMMT. However, this is not surprising as Laponite XLG has a much larger specific surface area (see Table 1). Since the interfacial interaction in polymer composites is proportional to the work of adhesion, which depends on the surface energy of the polymer and filler [43], we can suppose from the results that NaMMT can create a stronger interaction with starch compared to laponite. The interaction between the layers is also more substantial in the case of montmorillonite, which can influence the exfoliation.

### 3.2. Structure of the Nanocomposites

**Figure 2:**
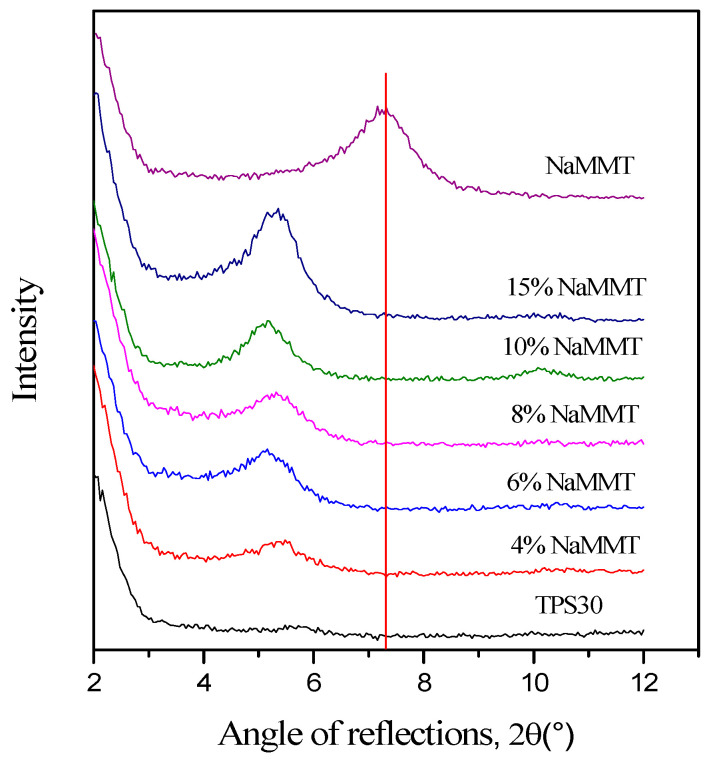
X-ray diffractograms of TPS30, NaMMT, and TPS30/NaMMT nanocomposites. The red line marks the maximum of the NaMMT diffraction peak indicated in the literature around 7.3°.

**Figure 3:**
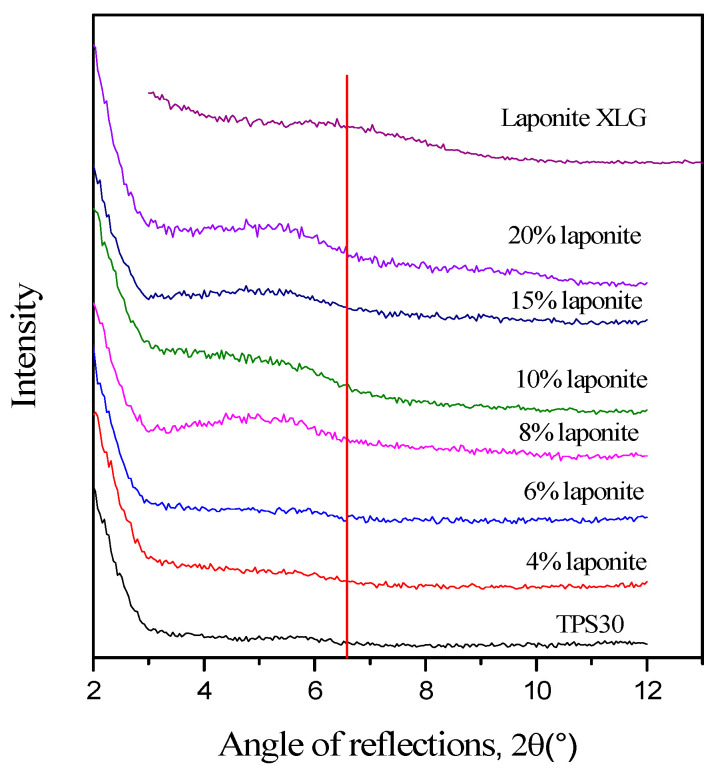
X-ray diffractograms of TP30, Laponite XLG, and TPS30/laponite nanocomposites. The red line marks the maximum of the laponite diffraction peak indicated in the literature around 6.6°.

**Figure 4:**
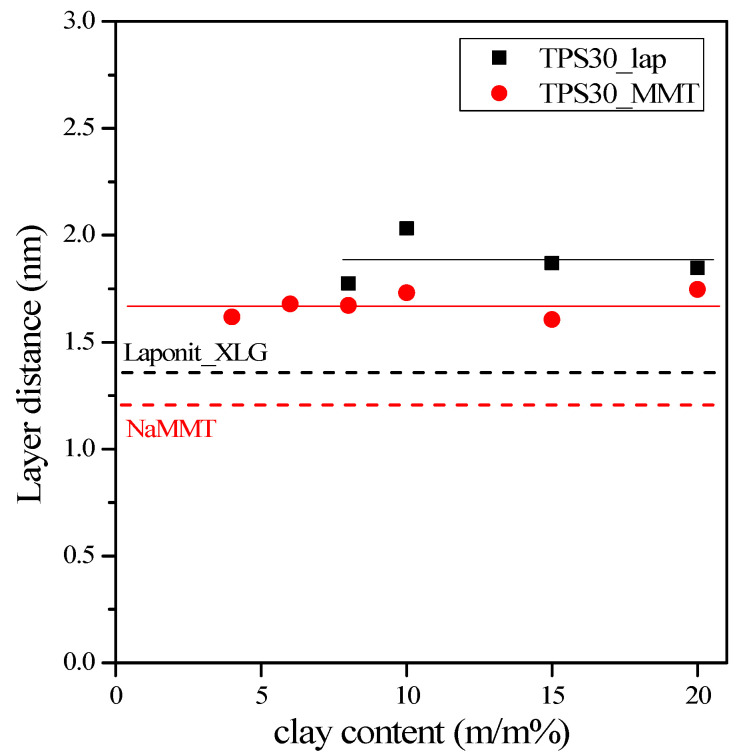
Dependence of the layer distance of silicates on the filler content in TPS30 nanocomposites.

The application of clays usually results in an intercalated/exfoliated structure in TPS nanocomposites. Figure 2 and Figure 3 show the XRD patterns obtained for the TPS nanocomposite films prepared by two different nanofillers (montmorillonite and laponite) and the XRD traces of the TPS and the clays. The 2θ range between 2° and 12° was analyzed to obtain information about the dispersion of the nanoclay in the TPS nanocomposites. Figure 2 shows the XRD patterns of NaMMT nanocomposites prepared by solution mixing at different NaMMT contents. Starch does not have any characteristic reflection in the studied range, while NaMMT exhibits a single 001 diffraction peak at around 7.3°. In the composite films, the 001 diffraction peak of NaMMT (1.20 nm) [16,19] shifts to smaller angles (5.3° ± 0.2°), corresponding to an interlayer basal spacing (d001) of 1.68 ± 0.06 nm independently of the clay content (Figure 4). The results are independent of the glycerol content of nanocomposites. These results indicate that either the glycerol, polymer chains, or both entered the silicate layers forming intercalated starch/MMT nanocomposites, without complete exfoliation. A mostly intercalated TPS/NaMMT structure with a 1.7–1.8 nm layer distance was detected in several papers [16,17,18,19,20]. The layer distance depended on the amounts of plasticizer and clay, as well as on the preparation method.

Although the diffraction peak of laponite is less intensive than that of montmorillonite, laponite XLG has a single diffraction peak at around 6.6° (Figure 3) [30] which shifts to smaller angles (4.8° ± 0.2°) in some nanocomposites. This means that the original layer distance of laponite (1.33 nm) is increased to 1.88 ± 0.1 nm in the TPS nanocomposites (see Figure 4). Figure 3 also indicates that the peak corresponding to the laponite reflection is practically absent at 4% and 6% laponite contents and shifts toward smaller 2-theta angles only at higher laponite contents. Exfoliation can be assumed at low clay concentrations and delamination at higher concentrations. Perotti [32] also observed that in TPS/laponite nanocomposite films prepared by film casting, the clay was completely exfoliated up to a 5% laponite content. At a laponite content higher than 5%, exfoliation was incomplete, with an intercalated laponite layer distance of between 1.8 and 2.2 nm. Aouada [33] showed that exfoliation was complete up to a 5% laponite content for composites prepared in an internal mixer. Kvien [31] found exfoliation up to a 3% laponite concentration for cast films.

**Figure 5:**
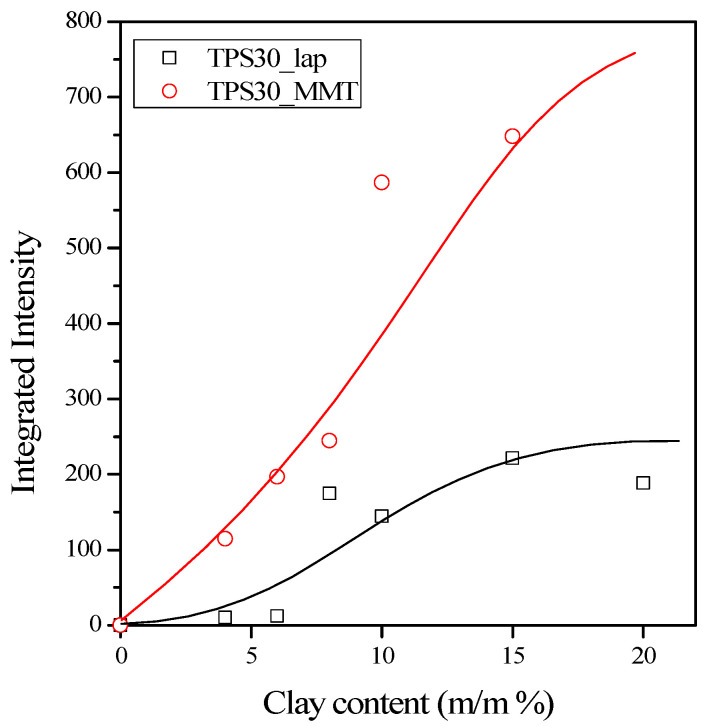
Integrated intensity of clay reflection plotted against the clay content.

The appearance of the silicate reflection with relatively high intensity for almost all nanocomposites indicates that the clays are not exfoliated, but intercalation or at most partial exfoliation may occur during processing. Figure 5 presents the dependence of the integrated intensity of silicate reflection on the clay content. The intensity increases almost linearly with the filler content in the case of NaMMT, which indicates that exfoliation does not occur during processing or always the same fraction of the silicate can exfoliates independently of the clay content. Dissimilarities in the gallery distance, order, and orientation may cause differences in the intensity of the various silicates. However, the different shapes of the integrated intensity curves of laponite and the relatively low intensities measured at low silicate contents suggest a slightly raised degree of exfoliation for this layered silicate.

Further information can be obtained about the structure of the composites from their light transmission. Examining TPS films by SEM at higher water contents is difficult; thus, the structure was studied with UV-VIS spectroscopy.

**Figure 6:**
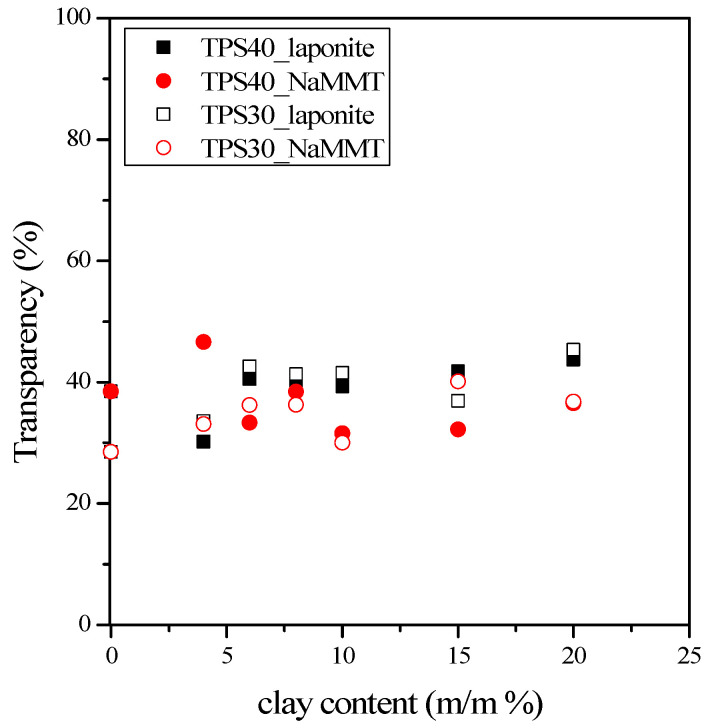
Effect of clay content on the transparency of different TPS/clay composites.

Composites containing particles smaller than the wavelength of incident light are transparent, while larger ones or aggregates scatter light and make the material opaque. Published results indicate that the transparency of nanocomposites increases with the increasing extent of exfoliation [44]. Figure 6 shows the transparency of the investigated nanocomposites as a function of the clay content. Except for some TPS30-NaMMT composites, the transparency of nanocomposites exceeds the transparency of TPS films, and it remains significant in the entire composition range, indicating the absence of microparticles. Although the transparency of TPS/laponite composites slightly exceeds that of TPS/NaMMT composites, it is difficult to draw a clear conclusion on the extent of exfoliation in these nanocomposites.

We must not overemphasize either the changes in gallery distance or transparency, but we can safely conclude that interactions occur among all components during the preparation of TPS composites. Complete exfoliation does not occur under the conditions used in this study, but intercalation and limited delamination cannot be excluded completely.

### 3.3. Mechanical Properties of Nanocomposites

**Figure 7:**
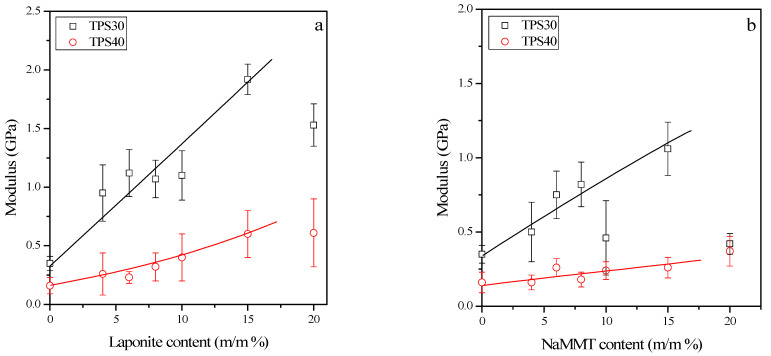

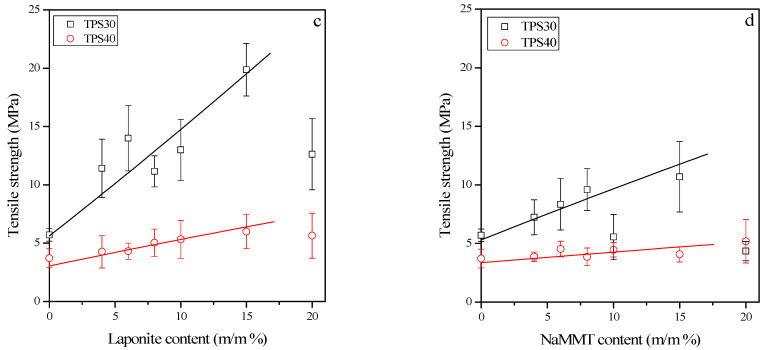
Mechanical properties: effect of laponite (**a**,**c**) and montmorillonite (**b**,**d**) content on the stiffness (**a**,**b**) and strength (**c**,**d**) of TPS/clay nanocomposites.

The stiffness, strength, and deformability of conditioned films were determined to characterize the mechanical behavior of the composites. The modulus and tensile strength values of TPS nanocomposites are plotted against the clay content in Figure 7.

Figure 7 unambiguously shows that the mechanical properties of the samples strongly depend on their plasticizer and clay content. The standard deviation of the measurements is relatively large even though the films showed a homogeneous appearance. The more substantial reinforcing effect of laponite can be clearly observed in the figures. In the case of laponite, both the modulus and tensile strength increase more significantly than they do for NaMMT when using the same amount of silicate. It can also be concluded that the nanofillers tend to aggregate when a high clay content is present (about 20%). Many researchers have demonstrated the stiffness and strength-enhancing effect of montmorillonite and laponite [18,20,22,24,28,29,31,33] in both cast and thermomechanically produced nanocomposites.

### 3.4. Reinforcing Effect of Clays

At large deformations, mechanical properties such as the tensile strength are determined by the composition, matrix properties, structure, and interactions. Interfacial interactions considerably influence and occasionally determine the properties of all heterogeneous materials. They also play an important role in the studied TPS/clay composites. Reinforcement, or the strength of interaction, can be estimated from the composition dependence of the tensile strength of composites. The use of a simple semi-empirical model developed earlier [45] allows us to calculate a parameter (B) that is proportional to the load carried by the dispersed component. The model takes the following form for tensile strength:

(2) $$\sigma_{Tred}=\sigma_{T\;}\frac{1+2.5\;\varphi }{1-\varphi \;}\;\frac{1}{\lambda^{n}}=\sigma_{Tm}\exp\left(B\varphi \right)$$

where σ_Tred_ is the reduced tensile strength, and λ is the extension ratio applied for the calculation of true tensile strength (σ_T_ = σλ). Eventually, n is a parameter taking into account strain hardening, and φ is the volume fraction of the filler. Equation (2) can easily be transformed into a linear form by taking the logarithm of both sides:

(3) $$\ln\sigma_{Tred}=\ln\;\left(\sigma_{T}\;\frac{1+2.5\;\varphi }{1-\phi }\;\frac{1}{\lambda^{n}}\;\right)=ln\sigma_{Tm}+B\;\varphi$$

The slope of this linear equation provides parameter B, which is proportional to the extent of reinforcement and determined by the specific surface area of the filler, the strength of interfacial interaction, and the matrix strength [43,45].

**Figure 8:**
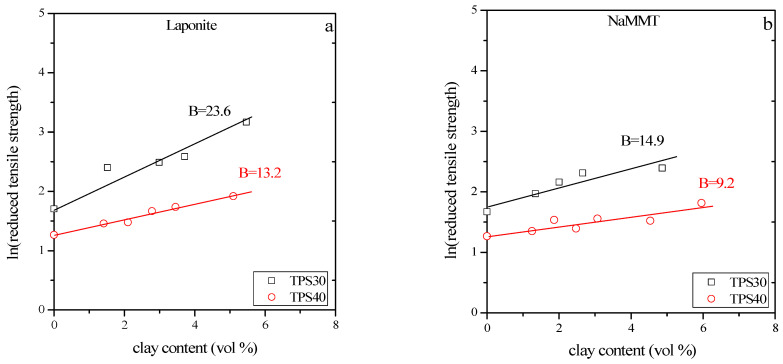
Reduced tensile strength of TPS nanocomposites plotted against filler content; (**a**) TPS/laponite, (**b**) TPS/montmorillonite.

In Figure 8, the reduced tensile strength of the composites is plotted against the filler content in the form indicated by Equation (3). Relatively good straight lines are obtained for the four selected cases, where standard deviation accounts for the scatter. The slope of the lines gives B the values listed in Table 4 together with the goodness of the linear fit, i.e., determination coefficients.

From Figure 8 and Table 4, we can conclude that parameter B is much larger in the case of laponite than montmorillonite. As we mentioned before, parameter B is larger if the interaction between polymer and filler is stronger and if the specific surface area of the filler is larger. In the case of nanocomposites, this area can be extremely large if significant exfoliation occurs. When comparing the surface energies (Table 3) of the clays, higher B values could be supposed in TPS/NaMMT composites. However, considering the higher specific surface area of laponite (Table 1), a higher B parameter can be assumed in TPS/laponite nanocomposites. The experimental results proved the latter assumption. The smaller surface energy of laponite, and the weaker interaction between the plates, can result in more significant exfoliation in TPS/laponite nanocomposites. XRD patterns confirmed (Figure 3 and Figure 5) the existence of exfoliation, and the considerable number of exfoliated layers led to improved stiffness and strength. Although the goodness of the linear fit of TPS30 nanocomposites was slightly less than 0.9, the calculated tensile strength of TPS matrix was very close to the measured values, proving the goodness of the fit.

### 3.5. Water Vapor Permeability of TPS Nanocomposites

**Figure 9:**
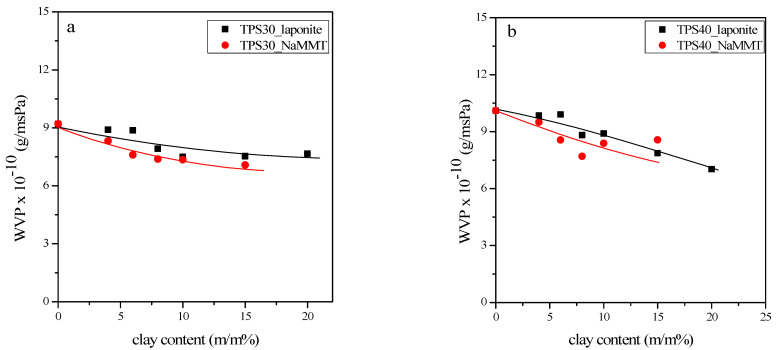
Dependence of water vapor permeability on clay content in TPS30 (**a**) and TPS40 (**b**) nanocomposites.

In addition to the importance of appearance and mechanical properties, water permeability is also essential for packaging materials. It is well-known that the strong hydrophilic character of TPS results in high water permeability. During the investigation, the effects of laponite and montmorillonite on WVP were studied. The results determined for 100–50 RH % are presented in Figure 9. The results clearly show that both the plasticizer and filler content affect the water vapor permeability, but neither effect is significant. The decrease in WVP is slightly larger for montmorillonite than for laponite, presumably due to the stronger polymer/filler interaction and the lower specific surface area of montmorillonite.

Only a limited number of publications in the literature provide data on the water vapor permeability of TPS/NaMMT [22,46,47] and TPS/laponite [32] nanocomposites. Although the published data are consistent with our results in terms of the order of magnitude, direct comparison of the WVP data is meaningless because the plasticizer and water content of the nanocomposites are different, and the WVP values were determined at various humidity differences.

## 4. Conclusions

We investigated the effect of particle characteristics and surface properties of laponite and sodium montmorillonite on the structure and properties of TPS nanocomposites. TPS/clay nanocomposites in a wide composition range and using two different concentrations of glycerol content were prepared by film casting. The XRD spectra and light transmission measurements showed that all TPS/NaMMT nanocomposites possess an intercalated structure. In TPS/laponite nanocomposites, effective exfoliation can also be supposed mainly at low clay contents. The small surface energy of laponite (153 mJ/m^2^) can facilitate the delamination of laponite layers. Thus, exfoliation further increases the already significant specific surface area of Laponite XLG clay. Although due to the larger surface energy of NaMMT (229 mJ/m^2^), stronger polymer/clay interaction and better mechanical properties could be assumed of TPS/NaMMT composites, the results proved that the better exfoliation and the much larger specific surface area of laponite result in higher reinforcement in TPS nanocomposites (the tensile strength of TPS30 increased from 5.6 MPa to 8.4 and 13.8 MPa with the addition of 5% montmorillonite and laponite, respectively). This was proven by the model of composition dependence of tensile strength. The TPS/laponite nanocomposites with better mechanical properties can act as excellent biodegradable packaging materials in addition to or instead of TPS/montmorillonite nanocomposites.

## Figures and Tables

**Table 1:** Characteristics of Nanofil 116 and Laponite XLG fillers.

	Nanofil 116	Laponite XLG
Density (g/cm^3^)	2.86	2.53
Diameter (nm)	240	25–30
Height (nm)	0.96	0.92
Water content (%)	8–13	<10
CEC (cation exchange capacity) (meq/100 g)	118	56
Specific surface (BET, N) * (m^2^/g)	42	388
Characteristic XRD peak (2Θ)	7.30	6.64
Gallery distance (nm)	1.21	1.33

* according to [41].

**Table 2:** Composition and designation of the TPS nanocomposites.

Clay	Glycerol Content (%) *	Clay Content (%) *	Designation of TPS Samples
Montmorillonite (NaMMT)	30	0, 4, 6, 8, 10, 15, 20	TPS30
Montmorillonite (NaMMT)	40	0, 4, 6, 8, 10, 15, 20	TPS40
Laponite XLG	30	0, 4, 6, 8, 10, 15, 20	TPS30
Laponite XLG	40	0, 4, 6, 8, 10, 15, 20	TPS40

* Based on the total amount of dry starch.

**Table 3:** Results of the IGC measurement.

Clay	Retention Times of n-Alkanes (s)			Flow Rate (cm^3^/min)	γ_S_^d^ (mJ/m^2^)
	Pentane	Hexane	Heptane		
Laponite	1.85 ± 0.17	8.79 ± 0.69	37.50 ± 1.37	66	153.1 ± 5.8
NaMMT	0.30 ± 0.01	1.97 ± 0.10	11.78 ± 0.14	57	229.4 ± 5.4

**Table 4:** Effect of matrix polymer and clay type on reinforcement.

Sample	Parameter B	R^2^	Tensile Strength of Matrix Polymer (MPa)	
			Measured	Calculated
TPS30-laponite	23.6	0.8960	5.70 ± 0.54	6.21
TPS40-laponite	13.2	0.9782	3.71 ± 0.80	3.53
TPS30-NaMMt	14.9	0.8515	5.70 ± 0.54	5.91
TPS40-NaMMT	9.2	0.8954	3.71 ± 0.80	3.54

## Data Availability

Data are contained within the article.
